# Burden of care among caregivers of people with mental illness in Africa: a systematic review and meta-analysis

**DOI:** 10.1186/s12888-024-06227-8

**Published:** 2024-11-07

**Authors:** Fantahun Andualem, Mamaru Melkam, Gebresilassie Tadesse, Girum Nakie, Techilo Tinsae, Setegn Fentahun, Gidey Rtbey, Girmaw Medfu Takelle, Getachew Muluye Gedef

**Affiliations:** 1 Department of Psychiatry, College of Medicine and Health Science, University of Gondar, PO Box 196, Gondar, Ethiopia; 2 Department of Psychiatry, School of Medicine, College of Medicine and Health Science, University of Gondar, Gondar, Ethiopia; 3 Department of General Midwifery, College of Medicine and Health Science, University of Gondar, Gondar, Ethiopia

**Keywords:** Prevalence, Burden of care, Family burden, Associated factors, Mental illness, Africa

## Abstract

**Background:**

Caring for people with mental illness requires a significant investment of personal physical, mental, social, and financial resources, which greatly impact the daily lives of caregivers. The process of providing care is multifaceted and intricate, involving both positive and negative emotional responses. Burden of care is a term used to describe the negative effects of caregivers’ burden on their physical, psychological, social, and economic well-being. Therefore, the aim of this systematic review and meta-analysis is to provide an overview of the most recent information available regarding the pooled prevalence of burden of care among people with mental illness in Africa.

**Methods:**

In this study, we followed the Preferred Reporting Items for Systematic Reviews and Meta-Analyses (PRISMA), which is a suitable guideline for reports of systematic reviews and meta-analyses. The PROSPERO protocol number for this review is CRD42024499138. To find publications for the systematic review and meta-analysis, we used PubMed, MEDLINE, EMBASE, Cochrane Library, and Scopus databases. The Joanna Briggs Institute (JBI) for cross-sectional study quality assessment was employed to evaluate the methodological quality of the studies included in this review. The data was extracted in Microsoft Excel, and then it was exported into STATA 11.0 for analysis. A funnel plot and an objective examination of Egger's regression test were used to check for publication bias.

**Results:**

We have included 12 studies conducted in African countries with 2156 study participants, of whom 1176 (54.55%) were female individuals. In this meta-analysis, the pooled prevalence of burden of care among caregivers of people with mental illness in Africa was 61.73 (95% CI: 51.25–72.21%). Further, in subgroup analysis regarding the study country, the pooled prevalence of carer burden among caregivers of people with mental illness in Egypt and Nigeria was 79.19% and 55.22%, respectively.

**Conclusion:**

This review found a high pooled prevalence of caregiver burden related to mental illness in Africa. To minimize the challenges faced by individuals with mental illnesses, as well as the burden on their caregivers, stakeholders may find these findings useful for addressing prevention, early screening, and management.

**Supplementary Information:**

The online version contains supplementary material available at 10.1186/s12888-024-06227-8.

## Introduction

All around the world, mental illnesses are very common and are a significant cause of disability and social burden [1]. Mental diseases have a detrimental impact on one's quality of life and general wellbeing, interfering with daily activities such as relationships, employment, and education [1, 2]. More than 20% of patients seen by primary health care professionals currently suffer from one or more mental illnesses. According to World Health Organization (WHO) estimates, at least one member of one in four families globally suffers from a mental illness [1]. Mental illnesses account for 5% of all diseases and 19% of all disabilities in Africa [3].

Over the past 60 years, there has been a gradual transition in the field of mental health care from hospital-based treatment to community-based treatment for individuals with mental illness [2, 4, 5]. Caregivers, including family, relatives, friends, voluntary and non-governmental organisations, and religious groups, are accountable for providing care for people suffering from mental illness. But when it comes to patient care and preventing readmission, family members (parents, siblings, and spouses) are the most crucial players [6, 7]. The WHO recognised that families were the primary providers of care for people with mental illnesses [8]. The family as a support system has been thoroughly examined, even if the abilities required of family caregivers to support patients' care have not been adequately established as a concept in nursing [9]. The study emphasised how support networks might shield people from psychological suffering [10]. Family caregivers suffer when mental health practitioners neglect to include them in the management and care plan for mentally ill relatives [11].

Caring for people with mental illness requires a significant investment of personal physical, mental, social, and financial resources, which greatly impact the daily lives of caregivers [12, 13]. In India, the carer burden scores for mental health patients were considerably greater than those for individuals with long-term medical conditions [14]. According to a systematic review study conducted in Africa, five out of seven respondents (71%) accounted for the total financial burden that serious mental illness places on carers [15]. The process of providing care is multifaceted and intricate, involving both positive and negative emotional responses [16]. Burden of care is a term used to describe the negative effects of caregivers’ burden on their physical, psychological, social, and economic well-being [12, 13, 17, 18].

There are two types of burden of care: objective and subjective. Objective burden is defined as the externally measured resources, such as time and money, that the carer devotes to providing care, whereas subjective burden is concerned with the carer's perception of the burden of care [19]. Caring for individuals with severe mental disorders is significantly more burdensome than caring for long-term physical diseases like diabetes and heart, kidney, or lung diseases, involving disruptions in family relationships, financial difficulties, and negative physical health effects [1].

The study found that carers of individuals with severe mental illness face a significant burden due to factors such as age, gender, education, health status, knowledge of mental illness, and environmental factors [17]. A substantial body of literature revealed that many factors, such as the length of the patient's illness [17], the patient's symptoms and diagnosis, their social support network, their financial resources [20, 21], the number of hospitalisations [21], their age, sex, and their educational status [22], are linked to the burden of care. According to other research, the length of illness and the frequency of hospitalisations are the factors most commonly linked to the burden of caregiving [21, 23].

According to a systematic review and meta-analysis, the overall pooled prevalence of carer burden among those who care for people with mental illness was 31.67% [24]. But no evaluation of carer burden among Africans caring for individuals with mental illness has been conducted. In fact, a great deal of research has been conducted on the burden that mentally ill patients place on their carers; stated prevalence rates range widely, from 33.7% [25] to 85.3% [26]. Another review study stated that carer burden was a risk factor for depressive symptoms among the carers of individuals with mental illness that can lead to the onset of clinical depression [27]. Caregivers of people with severe mental illness may encounter a number of distressing scenarios as the illness progresses [28]. There is a significant carer burden, particularly in developing nations where public health and medical resource constraints are more prevalent [29, 30].

Carers are essential in supporting those who suffer from mental illness. Reducing carers' workload and enhancing their psychological well-being could enable them to continue supporting others and manage the difficulties of caregiving [31]. Our search revealed that, among African carers of individuals with mental illnesses, there has not been a comprehensive study or meta-analysis of the prevalence of carer burden and its associated characteristics. Therefore, the aim of this systematic review and meta-analysis is to provide an overview of the most recent information available regarding the pooled prevalence of burden of care among caregivers of people with mental illness in Africa. To close this research gap, the following questions will be addressed:

1. What is the prevalence of carer burden among caregivers of people with mental illness?
2. What is the prevalence of carer burden by study country, care recipients’ mental illness, domain of carers, and assessment tool?

## Materials and methods

### Protocol

In this study, we followed the Preferred Reporting Items for Systematic Reviews and Meta-Analyses (PRISMA) [32] (Supplementary File 1), which is a suitable guideline for reports of systematic reviews and meta-analyses. The PROSPERO protocol number for this review is CRD42024499138. The criteria for the meta-analysis of observational studies in epidemiology [33] were also followed.

### Sources and data search strategy

The PICOT technique was used to design the search strategy for this systematic review in the manner described below [34]: The P (population of interest) consisted of carer burden among Africans who care for individuals with mental illness. For this review, intervention (I) and comparison (C) control groups were not necessary. The outcome (O) was measured using the carer burden among carers of individuals with mental illness assessment tools. The study topic (T) was determined by looking at all empirical studies that published primary data relevant to the study themes. To find publications for the systematic review and meta-analysis, we used both manual and electronic searches. The publications were searched by PubMed, MEDLINE, EMBASE, Cochrane Library, and Scopus databases. A search strategy was developed for the database by using a combination key term and Medical Subject Headings (Mesh). We used the following search key terms (prevalence OR epidemiology OR magnitude AND "burden of care" OR "family burden" OR “relative burden” OR “affiliate burden” AND "associated factors" OR "risk factors" AND "patients with mental illness" OR “psychiatric patients” AND Africa). Supplementary File 2 presents the full electronic search strategy developed with the assistance of a medical librarian. The search was conducted from January 1, 2024, to February 22, 2024.

### Eligibility criteria

#### Included criteria

Observational studies that report prevalence and/or associated factors of burden of care among caregivers of people with mental illness that were published in peer-reviewed journals in English were included. The studies, which were conducted on the continent of Africa and published from January 1, 2000, to February 22, 2024, were included in this systematic review and meta-analysis.

#### Exclusion criteria

Studies were excluded if they reported prevalence and/or associated factors of burden of care among caregivers of children (under the age of 18) with neurodevelopmental disorders or mental illness, conference abstracts, case reports, reviews, commentaries, and grey papers. Besides, studies without access to the full data and duplicated studies were also excluded.

### Study screening and selection

The searching, screening, and article selection were carried out independently by the two authors, FA and GMG. Importing research papers into EndNote X20 from the approved databases and removing duplicates was the first stage. The entire texts of the articles were read after any irrelevant ones were eliminated based on an evaluation of their abstract and title. Two authors (FA and GMG) conducted a cross-check after the searches. When searching, filtering, and selecting the articles, the two authors reached differing conclusions, which they then discussed with another author (GR) before reaching a consensus.

### Quality assessment

The Joanna Briggs Institute (JBI) for cross-sectional study quality assessment was employed to evaluate the methodological quality of the studies included in this review [35]. Using JBI, the two authors, SF and GR, separately assessed the original research's quality. Papers that scored five or higher on a nine-point scale were included in this review for analysis. The instrument has a total of nine ratings.

### Data extraction

When the papers met the eligibility criteria and quality of assessment, the data was extracted using Microsoft Excel. The two authors, TT and GT, independently extracted all the necessary data from the articles using a standardized data extraction format. The data extraction format included the following items: The first author's name, the publication year, the country in which the study was conducted, care recipients’ illness (the domain of mental illness of people with mental illness), participants relation to care recipients (caregivers’ relationship with mental ill patients), study design, study setting, burden of care assessment tool, the number of participants, the prevalence of burden of care, the score of quality assessment, and associated factors were all included in the data extraction format.

### Data analysis and publication bias

FA computed the logarithm and standard error of the logarithm of the prevalence to investigate the prevalence of burden of care among the included studies. Regarding associated factors, odds ratios, the logarithm of the odds ratio, and the standard error of the logarithms of the odds ratio were calculated. The data was extracted in Microsoft Excel, and then it was exported into STATA 11.0 for analysis. Summaries of the data were displayed using the random analysis effects model, and the Q and I^2^ tests were employed to look at study heterogeneity [36]. The low, moderate, substantial, and high heterogeneities were denoted by the ≤ 25%, 25–50%, 50–75%, and ≥ 75% I^2^ heterogeneity thresholds, respectively [37, 38]. The meta-analysis and narrative analysis were used to describe the results. The pooled prevalence was described using percentage and 95% confidence interval as summary statistics. A leave-one-out meta-analysis was used for sensitivity analysis. Sensitivity analysis was used to check whether overall finding was robust to potentially influential decisions. An estimate of random variation across studies, which is the foundation of the random effects model, was used. A funnel plot [39] and an objective examination of Egger's regression test [40], were used to check for publication bias. If the Egger's regression assumption test result was statistically significant (*p* < 0.05) or the funnel plot was asymmetrical, publication bias was reported [39, 40]. The study country, care recipients’ illness, participants relation to care recipients, study design, study setting, burden of care assessment tool were all the subjects of a subgroup analysis.

## Results

### Description of included studies

**Fig. 1:**
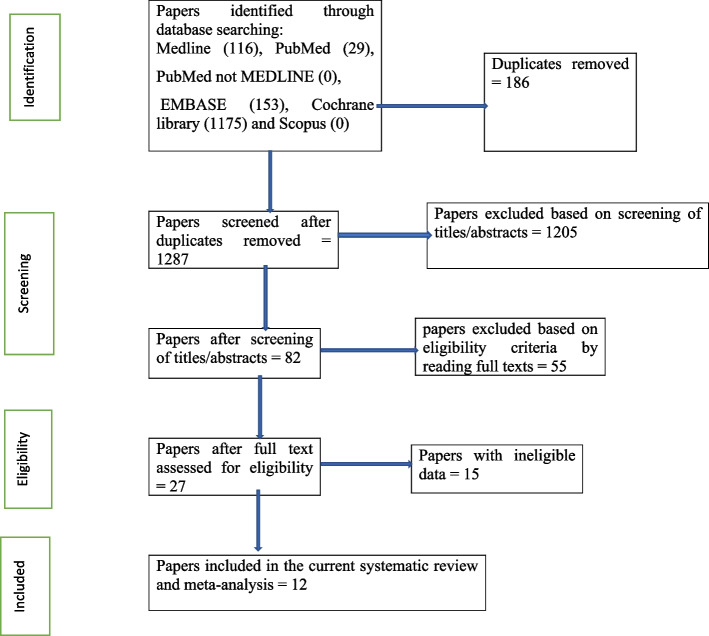
PRISMA flowchart of review search on the prevalence of burden of care among caregivers of people with mental illness in Africa

The systematic literature search identified a total of 1473 papers. Of these, 186 duplicate papers were eliminated, leaving 1287. After checking the titles and abstracts, 1205 papers were excluded. Following this screening, 82 articles remained. Of these, 55 were excluded based on eligibility criteria after full-text reviews, leaving 27 papers. Then, 15 papers were excluded due to ineligible data. Finally, this systematic review and meta-analysis included a total of 12 papers (Fig. 1).

### Study quality assessment

The Joanna Briggs Institute's (JBI) quality assessment criteria were used to evaluate the papers' quality. All articles involved in this review have good quality (JBI score ≥ 5) (Supplementary File 3).

### Characteristics of included studies

We have included 12 studies conducted in African countries with 2156 study participants, of whom 1176 (54.55%) were female individuals. Twelve studies were selected, half of which were carried out in Nigeria [25, 26, 41–44], three in Egypt [45–47], one each in Ethiopia [48], Tanzania [49], and Ghana [50]. With regard to the care recipients’ mental illness, seven studies were conducted on patients with schizophrenia, each two studies focused on severe mental illness in general and on patients with mental illness in general, and one study focused on bipolar affective disorders. Based on participants (caregivers) in relationship to the care recipients or people with mental illness: the caregivers either family, distant relative or not-relative participated in 7 studies; family caregivers participated in 4 studies; and family and distant relative participated in one study. Based on the assessment tool, six studies were assessed using the Zarit Burden Interview (ZBI) [51], three studies were assessed using the Family Burden Interview Schedule (FBIS) [52], two studies were assessed using the Burden Assessment Schedule (BAS) [53], and one study was assessed using the Burden of Care (BOC) Schedule [54] (Table 1).

**Table 1:** Characteristics of included studies on burden of care among caregivers of people with mental illness in Africa

First author name, year	Country	Care recipients’ illness (the domain of mental illness of people with mental illness)	Participants (caregivers) relationship to care recipients	SD	SS	Tool	SZ	P, %	PF, %	QA	AFW:AOR (95% CI)
Ahmed R, 2021 [45]	Egypt	Severe mental illness	Family caregivers: Parents, 36 (51.4%) Siblings, 22(31.4%) Spouse, 12(17.2%)	CS	PSU	ZBI	70	61.4	67.1	7	…
Amira AS, 2023 [46]	Egypt	Schizophrenia	Family and distant relative caregivers: Parents, 33(33%) Siblings, 34(34%) Others^a^, 33(33%)	CS	PSU	ZBI	100	82	18	6	…
Caroline EO, 2023 [41]	Nigeria	Severe mental illness	Family, distant relative and not relative caregivers: Parents, 54(38.3%) Siblings, 18(12.8%) Spouse, 53(37.6%) Others^b^, 13(11.3%)	CS	PSU	ZBI	141	37.6	56.7	7	…
Chidi JO, 2021 [42]	Nigeria	Bipolar affective disorders	Family, distant relative and not relative caregivers: Parents, 54(54%) Siblings, 13(13) Children, 5(5%) Spouse, 23(23%) Others^b^, 5(5%)	CS	PSU	FBIS	100	79	57	6	…
Chukwuweta CO, 2023 [43]	Nigeria	Schizophrenia	Family, distant relative and not relative caregivers: Parents, 65 (52.8%) Siblings, 33(26.8%) Children, 7(5.7%) Spouse, 6(52.8%) Others^b^, 12(9.8%)	CS	PSU	ZBI	123	49.2	50	7	…
Dominic U, 2011 [25]	Nigeria	Schizophrenia	Family, distant relative and not relative caregivers: Was not report separately	CS	PSU	ZBI	101	33.7	58.4	7	…
Mohammed A, 2019 [48]	Ethiopia	Mental illness	Family, distant relative and not relative caregivers: Parents, 156(38.4%) Siblings, 130(32%) Children, 56(13.8%) Spouse, 43(10.6%) Others^b^, 21(5.2%)	CS	PSU	FBIS	406	72.9	45	7	Stigma of caregivers: 2.69(2.37, 3.01)
Mona SH, 2023 [47]	Egypt	Schizophrenia	Family caregivers: Parents, 90(48.5%) Siblings, 52(28%) Children, 12(6.5%) Spouse, 32(17.2%)	CS	PSU	BAS	186	79	53.8	7	…
Rosarito C, 2022 [49]	Tanzania	Schizophrenia	Family caregivers: Parents, 32(48.5%) Siblings, 12(18.2%) Children, 2(3%) Spouse, 7(10.6%) Others^a^, 13(19.7%)	CS	PSU	BAS	65	63.1	65.2	5	Family Functioning: 4.87(1.19, 19.25)
Victor OL, 2013 [26]	Nigeria	Schizophrenia	Family, distant relative and not relative caregivers: Parents, 219(59.5%) Siblings, 49(13.3%) Spouse, 43(11.7%) Others^b^, 57(15.5%)	CS	PSU	FBIS	368	85.3	78.3	7	…
Yaw NO, 2017 [50]	Ghana	Schizophrenia	Family caregivers: Was not reported separately	CS	PSU	ZBI	444	49	56.5	6	…
Yo O, 2012 [44]	Nigeria	Mental illness	Family caregivers: Parents, 19(38.8%) Siblings, 9(18.4%) Spouse, 8(16.3%) Others^a^, 13(26.5%)	CS	PSU	BOC	53	45.3	49	6	…

*SD *study design, *CS *cross sectional, *SD *study setting, *PSU *psychiatry service unit of health service institution, *SZ *sample size of the caregivers /participants, *P % *prevalence of caregivers’ burden, *PF % *percentage of female among the participants, *QA *quality assessment, *AFW *AOR (95% CI), associated factors with adjusted odd ratio in 95% confidence interval

^a^Others, distant biological relatives (uncle/aunt, grandparents, cousin and like)

^b^Others, distant biological relatives and non-biological care givers like friends and volunteer caregivers in non-governmental or religious charity center

### The pooled prevalence of burden of care

**Fig. 2:**
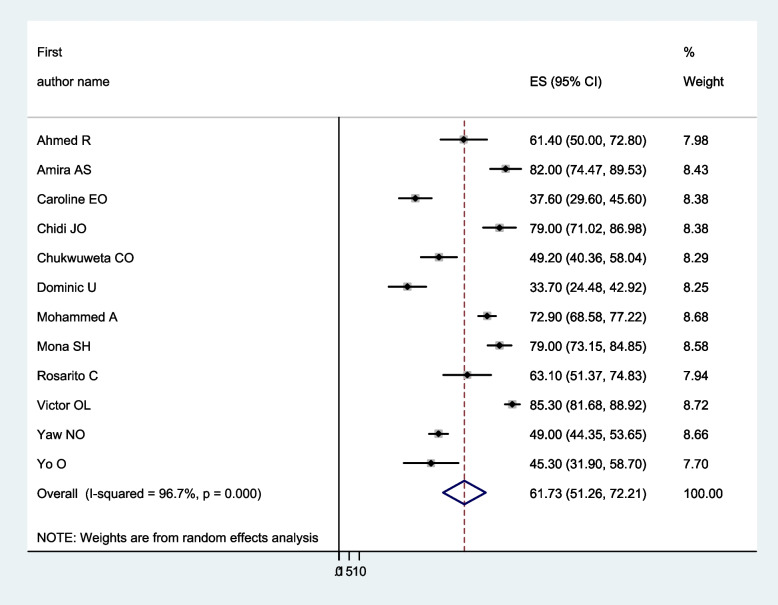
Forest plot of the pooled prevalence of burden of care among caregivers of people with mental illness in Africa

Figure 2 shows in this meta-analysis, the pooled prevalence of burden of car among caregivers of people with mental illness in Africa to be 61.73 (95% CI: 51.25–72.21%). Based on the apparent heterogeneity among the studies, we conducted a meta-analysis using a random effect model (I^2^ = 96.70%, *p* < 0.001).

### Subgroup analysis

**Fig. 3:**
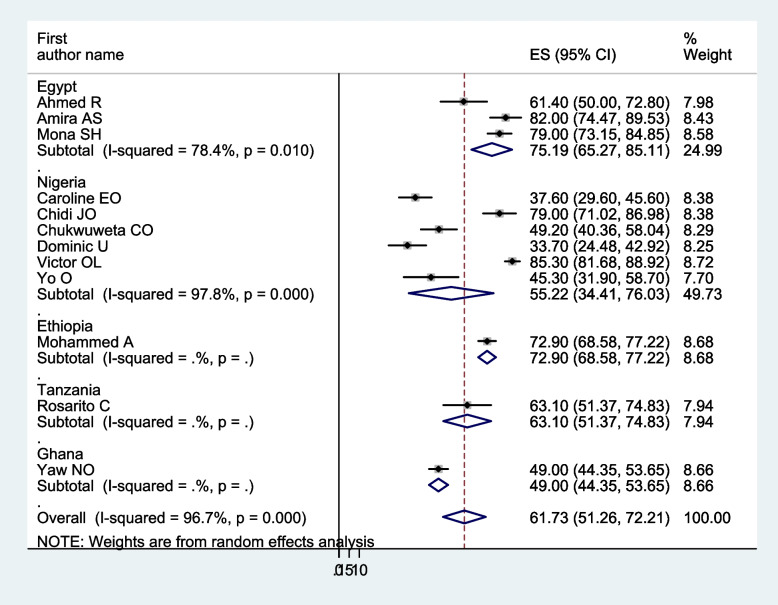
Forest plot, on subgroup analysis based on the study country, of the pooled prevalence of burden of care among caregivers of people with mental illness in Africa

**Fig. 4:**
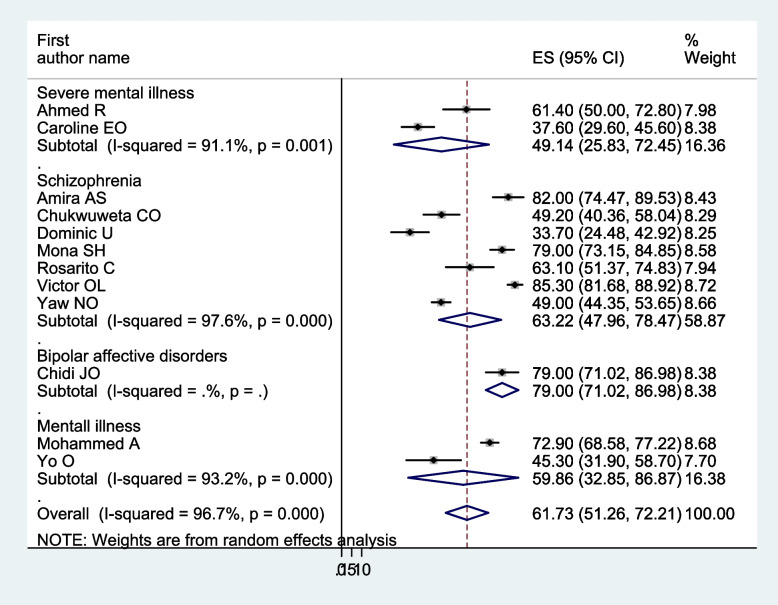
Forest plot, on subgroup analysis based on care recipients’ illness, of the pooled prevalence of burden of care among caregivers of people with mental illness in Africa

**Fig. 5:**
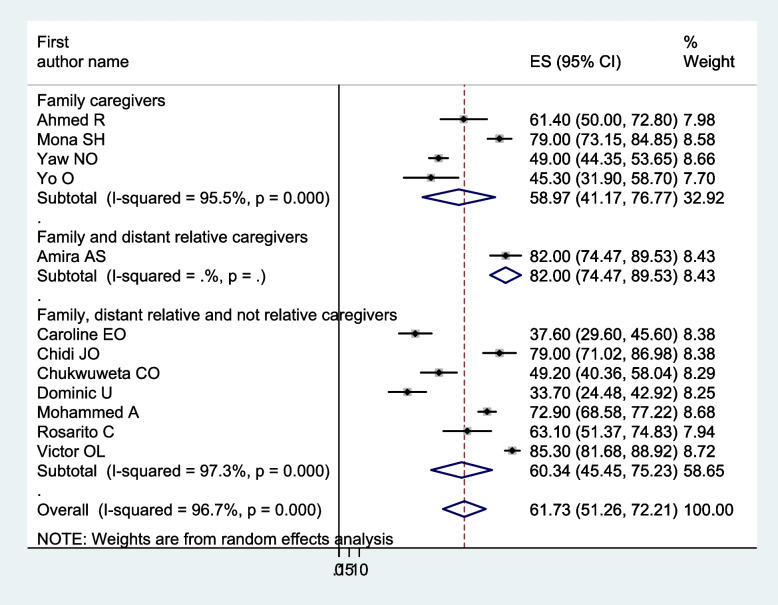
Forest plot, on subgroup analysis based on the domain of caregiver participants, of the pooled prevalence of burden of care among caregivers of people with mental illness in Africa

**Fig. 6:**
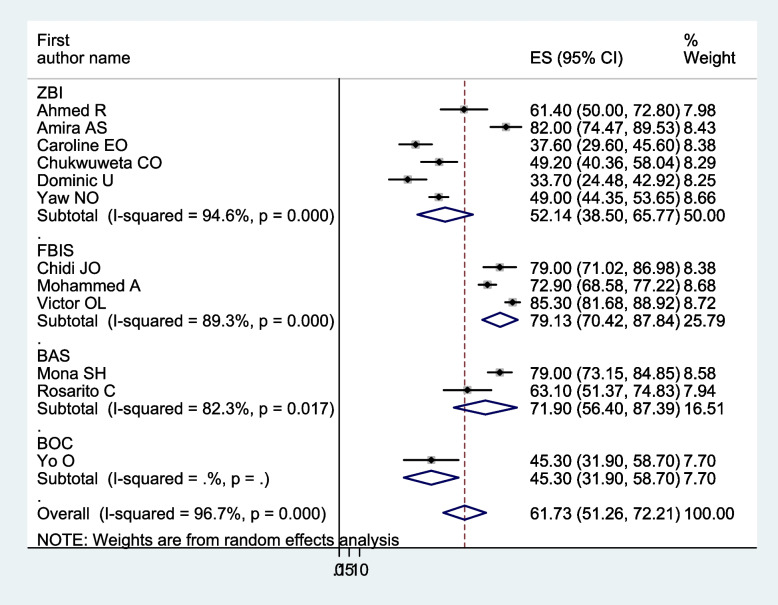
Forest plot, on subgroup analysis based on the assessment tool, of the pooled prevalence of burden of care among caregivers of people with mental illness in Africa

In a meta-analysis of the pooled prevalence of carer burden, heterogeneity was found (I^2^ = 96.70%, *p* < 0.001). Thus, a subgroup analysis was carried out: the study country, the mental illness of the care recipients, the relationship between the carers and those who had care recipients, and the assessment instrument. The highest pooled prevalence of carers’ burden of people with mental illness was found in Egypt, 79.19% (I^2^ = 78.40%, *p* < 0.01), and followed by Nigeria, 55.22% (I^2^ = 97.80%, *p* < 0.001). Based on the name of illness for care recipients, the pooled prevalence for patients with severe mental illness was 49.14% (I^2^ = 91.10%, *p* < 0.01), patients with schizophrenia was 63.22% (I^2^ = 97.60%, *p* < 0.001), and patients with mental illness in general was 59.86% (I^2^ = 93.20%, *p* < 0.001). Regarding carer relation to care recipients, family caregivers accounted for 58.97% (I^2^ = 95.50%, *p* < 0.001), and family, distant relative, and not relative carers accounted for 60.34% (I^2^ = 97.30%, *p* < 0.001). Another analysis was done based on the assessment tool; the highest pooled prevalence of carer burden among caregivers of people with mental illness, 79.13% (I^2^ = 89.3%, *p* < 0.001), was assessed by the FBIS. Of 52.14% (I^2^ = 94.60%, *p* < 0.001) and 71.90% (I^2^ = 82.30%, *p* < 0.017), the pooled caregivers’ burden of people with mental illness was measured using the ZBI and BAS, respectively (Figs. 3, 4, 5, and 6).

### Publication bias

**Fig. 7:**
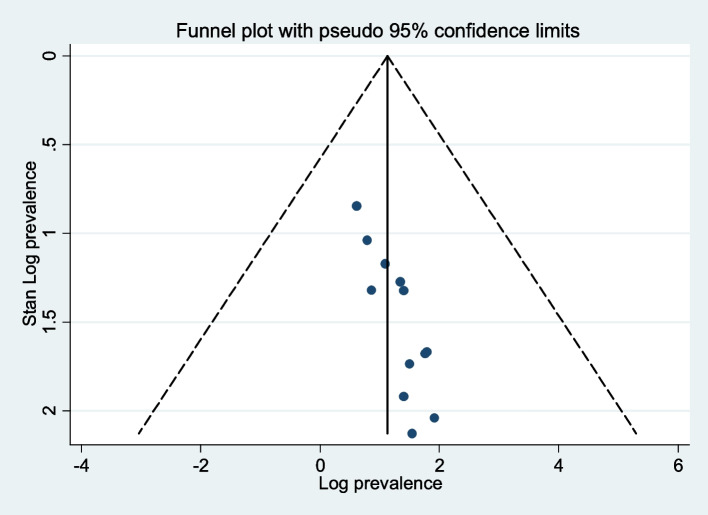
Funnel plot showing publication bias of prevalence of burden of care among caregivers of people with mental illness in Africa

The results of this study's funnel plot (Fig. 7) show that there is no publication bias, and Egger's regression test (*P* = 0.111) supported this finding (Table 2).

**Table 2:** Egger’s test of burden of care among caregivers of people with mental illness in Africa

Std_-_Eff	Coef	Std. Err	T	P > t	[95% Conf Interval]	
Slope	87.78797	12.03709	7.29	0.001	60.96765	114.6083
Bias	-6.524199	3.726575	-1.75	0.111	-14.82753	1.779128

### A leave-out-one sensitivity analysis

**Fig. 8:**
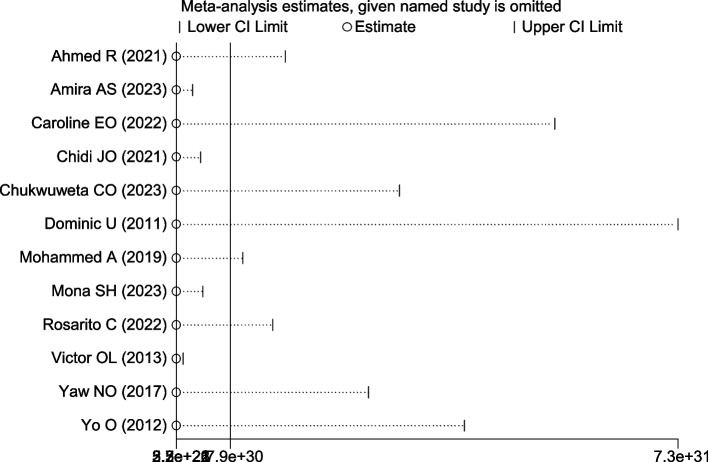
Sensitivity analysis of prevalence of burden of care among caregivers of people with mental illness in Africa, a study being removed at a time: prevalence and 95% CI (The analysis is based on Random effect model)

In this systematic review and meta-analysis study, the effect of each study on the pooled prevalence of carer burden among carers of people with mental illness was examined using a sensitivity analysis, which involved gradually eliminating one study at a time to test the heterogeneity of those findings. The prevalence of this systematic review and meta-analysis was not significantly affected by the omission of a single article, as shown by the results, which ranged from 59.35 to 64.04% (Table 3) and (Fig. 8).

**Table 3:** Sensitivity analysis of burden of care among caregivers of people with mental illness in Africa

Study omitted	Estimate 95% CI	Heterogeneity	
		I^2^ (%)	P- value
Ahmed R, (2021) [45]	61.53(51.21–71.85)	99.40	0.000
Amira AS, (2023) [46]	59.65(49.35–69.95)	99.40	0.000
Caroline EO, (2022) [41]	63.69(54.29–73.09)	99.30	0.000
Chidi JO, (2021) [42]	59.92(49.51–70.34)	99.40	0.000
Chukwuweta CO, (2023) [43]	62.64(52.72–72.56)	99.40	0.000
Dominic U, (2011) [25]	64.04(54.70–73.37)	99.30	0.000
Mohammed A, (2019) [48]	60.47(49.59–71.35)	99.40	0.000
Mona SH, (2023) [47]	59.92(49.42–70.42	99.40	0.000
Rosarito C, (2022) [49]	61.37(51.02–71.72)	99.40	0.000
Victor OL, (2013) [26]	59.35(49.67–69.03)	99.20	0.000
Yaw NO, (2017) [50]	62.67(52.92–72.42)	99.30	0.000
Yo O, (2012) [44]	62.99(53.16–72.82)	99.40	0.000

### Associated factors analysis

**Fig. 9:**
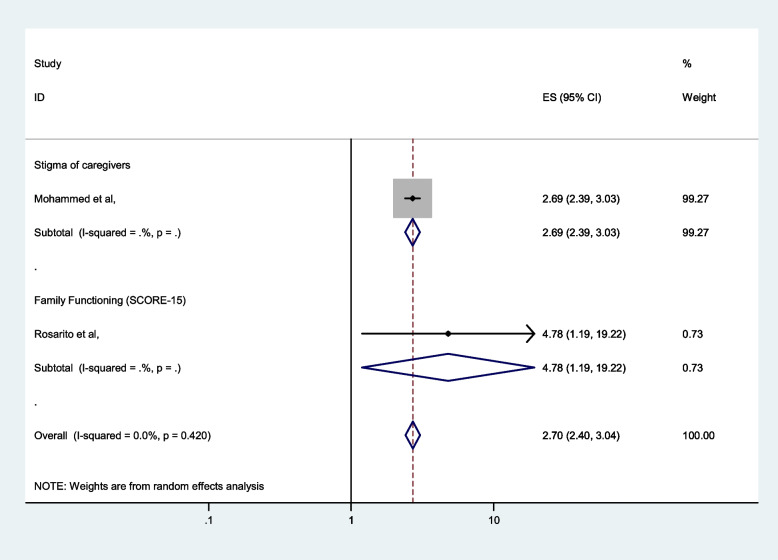
Forest plot showing different associated factors of burden of care among burden of care among caregivers of people with mental illness in Africa

In Table 1, we extracted important sociodemographic and other factors that are associated with carer burden cwith reference to the studies analysed in logistic regression with an adjusted odd ratio. Unfortunately, the individual papers found only two factors: the stigma of carers [48] and family functioning [47]. The pooled analysis was done to determine the pooled effect of the factors when the factors were associated with two or more papers. Thus, in this review, we limited our analysis to factors associated with carer burden among people with mental illness (Fig. 9).

## Discussion

The challenges faced by carers of individuals suffering from mental illness included an inability to meet their needs, burnout, heavy care pressure, a high level of social stigma, a shortage of social support, and a poor quality of life [6]. Using an adjusted odd ratio in logistic regression, important variables pertaining to the burden carers bear for individuals with mental illnesses were collected at the time of data extraction for each individual study. On the other hand, no significant variable that was associated with the difficulty of caring for two or more publications was found. Therefore, the purpose of this systematic review and meta-analysis is to provide an overview of the most recent information available regarding the pooled prevalence of carer burden among African carers of individuals with mental illness.

We synthesised 12 papers to examine the prevalence of carer burden among 2156 carers of individuals with mental illness for this systematic review and meta-analysis. Of these, more than half (1331) carers reported having a carer burden. In the current finding, the pooled prevalence of carer burden among carers of people with mental illness was 61.73 (95% CI: 51.25–72.21%). This finding was consistent with a previous study on carer burden among those who care for individuals with mental illness conducted in Iran [55], which involved systematic reviews and meta-analyses. Whereas it was higher than the carer burden among patients with mental illness, as determined in systematic reviews and meta-analyses conducted globally [56], and the family burden associated with mental and physical diseases, as reported in the WHO World Mental Health (WMH) surveys [57]. This discrepancy may be caused by differences in the residence of study participants, the study setting, participants, sample size, and the measurement tool. This analysis focused on five African nations, whereas the previous review included 39 studies from 23 different countries and examined the prevalence of carer burden among 5034 carer participants, of which only two countries (Nigeria and Egypt) are in Africa. Eleven studies were carried out in the community, while seventeen were carried out in a health care facility. Furthermore, a number of additional assessment instruments were employed, including the Family Burden Scale (FBS), the Perceived Chronic Strains Scale (PCSS), the Self-Perceived Pressure by Informal Care Scale (SPP-ICS), the Involvement Evaluation (IV), the Carer Strain Inventory (CBI), the Family Problems Questionnaire (FPQ), the Feetham Family Functioning Scale (FFFS), and the Social Behaviour Assessment Schedule (SBAS). A total of 2156 carers of individuals with mental illness participated in our systematic review and meta-analysis at health service institutions across five nations.

The World Mental Health Survey was a community-based epidemiological survey done in countries worldwide. Data from the 19 WMH surveys, which evaluated the family burden of mental and physical diseases, served as the basis for this report. The World Bank has categorised ten of these as high-income countries (Belgium, France, Germany, Israel, Italy, the Netherlands, Northern Ireland, Portugal, Spain, the United States), five as upper-middle income countries (Sao Paulo, Brazil; Bulgaria, Lebanon, Mexico, Romania); and four as low-income or lower-middle income countries (Colombia, Iraq, Nigeria, and Shenzhen in the People's Republic of China. The countries included in this study were grouped as low- or lower-middle-income countries. In the WMH survey, a total of 87,748 carers of mental and physical disorders, of those 43,732 respondents, experienced family burden across all the countries using the WMH Survey version of the Composite International Diagnostic Interview (CIDI 3.0) [58]. In this review, 12 studies investigating the prevalence of carer burden among 2156 carer participants were done in five countries in Africa. Of the 1331 carers of people with mental illness, 1331 experienced carer burden. The carer burden in this study was determined by various assessment instruments. For example, the Zarit Burden Interview (ZBI) was used to evaluate six studies. ZBI was originally created to measure the degree of burden that primary carers of elderly individuals with senile dementia and disabled individuals faced [51]; however, it has also been used in Africa to measure carer burden in cases of schizophrenia [59–61]. The Family Burden Interview Schedule (FBIS) was used to evaluate three studies. FBIS was initial developed in India by Pai and Kapur [52]. The interview schedule is semi-structured and evaluates both subjective and objective burdens. In Africa, a modified version of this FBIS was developed [62]. The Burden Assessment Schedule (BAS), which was developed and utilised by the researcher to evaluate the objective and subjective burden faced by those who care for patients with chronic mental illness, was used to assess two further investigations [53]. Additionally, the Burden of Care (BOC) Schedule was utilised in one study to determine the burden of care. The tool was first developed and used in an Israeli family-based research project [54]. It was also given to a different set of carers in order to assess its face validity and other psychometric qualities in advance of its application in a different study setting [44].

In the current study, regarding subgroup analysis, the pooled prevalence of carer burden among carers of people with a mental illness was 75.19% in Egypt, which was higher compared with 55.22% in Nigeria. This finding may vary depending on the number of studies, participant sample size, and assessment tool. For example, three studies with 356 participants were conducted in Egypt, and six studies with 781 participants were conducted in Nigeria. It could also be due to variations in research regions and assessment tools, along with sociocultural differences.

All things considered; our research reveals significant rates of carer burden that vary by country according to conditions. Mental illness may not be considered a life-threatening disease in several nations, including Ethiopia [63, 64]. As a result, mental health care and treatment may not be given priority by policymakers, planners, and healthcare professionals [65, 66]. For those impacted by these illnesses, inadequate understanding and priority-setting can play a major role in determining poor mental health outcomes. Therefore, expanding government mental health treatment services is crucial for the future. This includes outreach initiatives and campaigns to raise public knowledge of mental health issues, which may help reduce challenges and improve social outcomes for those who suffer from mental illness [67]. Furthermore, African nations should prioritise developing the capacity of policymakers and healthcare professionals, as well as strengthening the governance and mental healthcare systems. Thus, the goal of this study is to provide a more in-depth understanding and shed additional light on developing country research and practice.

### Strengths and limitations of the study

This research may offer new perspectives on managing the burden faced by caregivers of people with mental illness and highlight the widespread burden experienced by individuals with mental illnesses. However, the limitations of this review include its focus on research published only in English and the relatively small number of papers included. Additionally, most of the studies used different tools to assess caregiver burden, making it challenging to evaluate and summarize the findings.

## Conclusion

This review found a high pooled prevalence of caregiver burden related to mental illness in Africa. To minimize the challenges faced by individuals with mental illnesses, as well as the burden on their caregivers, stakeholders may find these findings useful for addressing prevention, early screening, and management. Additionally, this study may provide valuable information to concerned bodies, such as policymakers, administrators, and health professionals, on the importance of focusing on early screening and managing caregiver burden. The researchers recommended that future studies focus on research published in languages other than English, include a larger number of papers, and use the same tool for assessment.

## Supplementary Information

Supplementary Material 1.

Supplementary Material 2.

Supplementary Material 3.

## Data Availability

This published article and its supplementary information files include all data generated or analysed during this study.

## Abbreviations

BAS: Burden Assessment Schedule
BOC: Burden of Care
CBI: Carer Strain Inventory
CIDI: Composite International Diagnostic Interview
CRD: Central Registration Depository
FBIS: Family Burden Interview Schedule Family
FBS: Burden Scale
FPQ: Family Problems Questionnaire
FFFS: Feetham Family Functioning Scale
IV: .Involvement Evaluation
JBI: Joanna Briggs Institute
PCSS: Perceived Chronic Strains Scale
PICOT: Population/Patient, Intervention, Comparison, Outcome and Time
PRISMA: Preferred Reporting Items for Systematic Reviews and Meta-Analyses
PROSPERO: International Prospective Register of Systematic Reviews
STATA: Statistics and Data
SPP-ICS: Self-Perceived Pressure by Informal Care Scale
SBAS: Social Behaviour Assessment Schedule
WMH: World Mental Health World
WHO: Health Organization
ZBI: Zarit Burden Interview
