# Supplementary material for: Burden of care among caregivers of people with mental illness in Africa: a systematic review and meta-analysis
Source: BMC Psychiatry. 2024 Nov 7;24:778. doi: 10.1186/s12888-024-06227-8 (PMC11542449; doi:10.1186/s12888-024-06227-8)
Supplement: Supplementary file 2 — Supplementary Material 2. [file 12888_2024_6227_MOESM2_ESM.docx]

**Database searching strategies:**

**Cochrane library**

1175 **Cochrane Reviews matching**(prevalence OR epidemiology OR magnitude AND "burden of care" OR "family burden" OR “relative burden” OR “affiliate burden” AND "associated factors" OR "risk factors" AND "patients with mental illness" OR “psychiatric patients” AND Africa) in Title Abstract Keyword

**EMBASE**

(((('prevalence'/exp OR 'prevalence' OR 'epidemiology'/exp OR 'epidemiology' OR 'magnitude'/exp OR 'magnitude') AND ('carer burden'/exp OR 'carer burden') OR 'family burden'/exp OR 'family burden' OR 'relative burden' OR 'affiliate burden') AND 'associated factors' OR 'risk factors'/exp OR 'risk factors') AND 'patients with mental illness' OR 'psychiatric patients') AND ('africa'/exp OR 'africa') AND [embase]/lim = 153

**MEDLINE**

(((('prevalence' OR 'epidemiology' OR 'magnitude') AND 'carer burden' OR 'family burden' OR 'relative burden' OR 'affiliate burden') AND 'associated factors' OR 'risk factors') AND 'patients with mental illness' OR 'psychiatric patients') AND 'africa' AND [medline]/lim = 116

**PED MED**

Filters applied: Abstract, Free full text, Full text, Introductory Journal Articles, from 2000/1/1 – 2024/2/22

(((((((((((((((((((((((((((prevalence) OR (prevalence[MeSH Terms])) ) OR (epidemiology)) OR (epidemiology[MeSH Terms]))) AND (burden)) OR (burden [MeSH Terms])) OR (care)) OR (care[MeSH Terms])) OR ("relative burden")) OR ("relative burden"[MeSH Terms])) OR ("affiliate burden")) ) OR ("affiliate burden"[MeSH Terms])) AND ("associated factors")) OR ("associated factors"[MeSH Terms])) OR ("risk factors")) OR ("risk factors"[MeSH Terms])) AND ("patients with mental illness")) OR ("patients with mental illness"[MeSH Terms])) OR ("psychiatric patients")) OR ("psychiatric patients"[MeSH Terms])) AND (Africa)) OR (Africa[MeSH Terms]) = 29

**PubMed not MEDLINE**

((((prevalence OR epidemiology OR magnitude) AND 'burden of care' OR 'family burden' OR 'relative burden' OR 'affiliate burden') AND 'associated factors' OR 'risk factors') AND 'patients with mental illness' OR 'psychiatric patients') AND africa AND [pubmed-not-medline]/lim = 0
