# Supplementary material for: Burden of care among caregivers of people with mental illness in Africa: a systematic review and meta-analysis
Source: BMC Psychiatry. 2024 Nov 7;24:778. doi: 10.1186/s12888-024-06227-8 (PMC11542449; doi:10.1186/s12888-024-06227-8)
Supplement: Supplementary file 3 — Supplementary Material 3. [file 12888_2024_6227_MOESM3_ESM.docx]

Quality assessment

| First author name (year) | Q1 | Q2 | Q3 | Q4 | Q5 | Q6 | Q7 | Q8 | Q9 | Total score (9%) |
| --- | --- | --- | --- | --- | --- | --- | --- | --- | --- | --- |
|  |  |  |  |  |  |  |  |  |  |  |
| Ahmed R, (2021) | NA | Y | NA | Y | Y | Y | Y | Y | Y | 7 |
| Amira AS, (2023) | NA | Y | NR | Y | Y | Y | Y | Y | NR | 6 |
| Caroline EO, (2022) | NA | Y | N | Y | Y | Y | Y | Y | Y | 7 |
| Chidi JO, (2021) | NR | NR | NR | Y | Y | Y | Y | Y | Y | 6 |
| Chukwuweta CO, (2023) | NA | Y | NA | Y | Y | Y | Y | Y | Y | 7 |
| Dominic U, (2011) | NA | Y | NA | Y | Y | Y | Y | Y | Y | 7 |
| Mohammed A, (2019) | NA | Y | NA | Y | Y | Y | Y | Y | Y | 7 |
| Mona SH, (2023) | NA | Y | NA | Y | Y | Y | Y | Y | Y | 7 |
| Rosarito C, (2022) | NR | Y | NR | Y | NR | Y | Y | Y | NR | 5 |
| Victor OL, (2013) | NA | Y | NR | Y | Y | Y | Y | Y | Y | 7 |
| Yaw NO, (2017) | NR | NR | Y | Y | Y | Y | Y | Y | NR | 6 |
| Yo O, (2012) | NA | Y | NR | Y | Y | Y | Y | Y | NR | 6 |

**Key:** **Y**= Yes; **N**= No; **NR**= Not Reported, **NA**=Not Applicable

**Question codes:**

1. Was the sample frame appropriate to address the target population?

2. Were study participants sampled in an appropriate way?

3. Was the sample size adequate?

4. Were the study subjects and the setting described in detail?

5. Was the data analysis conducted with sufficient coverage of the identified sample?

6. Were valid methods used for the identification of the condition?

7. Was the condition measured in a standard, reliable way for all participants?

8. Was there appropriate statistical analysis?

9. was the response rate adequate, and if not, was the low response rate managed appropriately?
